# Freshwater Clam Extract Ameliorates Triglyceride and Cholesterol Metabolism through the Expression of Genes Involved in Hepatic Lipogenesis and Cholesterol Degradation in Rats

**DOI:** 10.1155/2013/830684

**Published:** 2013-02-12

**Authors:** Thomas Laurent, Yuji Okuda, Takeshi Chijimatsu, Miki Umeki, Satoru Kobayashi, Yutaro Kataoka, Iwao Tatsuguchi, Satoshi Mochizuki, Hiroaki Oda

**Affiliations:** ^1^Department of Applied Molecular Biosciences, Nagoya University, Furo-cho, Chikusa-ku, Nagoya 464-8601, Japan; ^2^Shizenshokken Co. Ltd., 293 Sakai, Bungotakada, Oita 879-0615, Japan; ^3^Sasaki Food Co. Ltd., 276 Sakai, Bungotakada, Oita 879-0615, Japan; ^4^Faculty of Education and Welfare Science, Oita University, 700 Dannoharu, Oita, Oita 870-1192, Japan

## Abstract

The freshwater clam (*Corbicula* spp.) is a popular edible bivalve and has been used as a folk remedy for liver disease in Asia. As a Chinese traditional medicine, it is said that freshwater clam ameliorates alcoholic intoxication and cholestasis. In this study, to estimate the practical benefit of freshwater clam extract (FCE), we compared the effects of FCE and soy protein isolate (SPI) on triglyceride and cholesterol metabolism in rats. FCE and SPI lowered serum cholesterol, and FCE tended to reduce serum triglycerides. FCE enhanced fecal sterol excretion and hepatic mRNA levels of *CYP7A1* and *ABCG5* more substantially than SPI; however, both diets reduced hepatic cholesterol. Both of the diets similarly suppressed liver lipids improved Δ9-desaturated fatty acid profile, and FCE was associated with a reduction in *FAS* and *SCD1* mRNA levels. Hepatic transcriptome analysis revealed that inhibition of lipogenesis-related gene expression may contribute to downregulation of hepatic triglycerides by FCE. FCE would have better potential benefits for preventing metabolic disorders, through greater improvement of metabolism of triglycerides and cholesterol, likely through a mechanism similar to SPI.

## 1. Introduction

Dietary factors are known to influence several risk factors for heart disease, including hypercholesterolemia, hypertriglyceridemia, elevated low-density lipoprotein cholesterol, reduced high-density lipoprotein cholesterol, diabetes, and obesity [1, 2]. Recently, this cluster of symptoms has been attributed to a specific condition: metabolic syndrome [3]. Among nonobese and type II diabetic subjects, hepatic steatosis, which principally reflects triglyceride accumulation, is a good predictor of serum triglyceride levels [4, 5]. Hepatic fat is correlated with secretion of very low-density lipoproteins (VLDLs) [6]. Hepatic triglycerides participate in VLDL assembly and are associated with insulin resistance [7].

The freshwater clam (*Corbicula *spp.) is a popular bivalve consumed as food in Asia. Freshwater clam has been also used as a folk remedy for liver disease in East Asia. In Compendium of Materia Medica (Ben Cao Gamg Mu), which is the complete and comprehensive Chinese traditional medical book, it is described that freshwater clam ameliorates alcoholic intoxication and cholestasis. Previously, we found that freshwater clam extract (FCE) improved hepatic and serum cholesterol levels in rats with diet- and xenobiotic-induced hypercholesterolemia through the gene expression of *CYP7A1*, involved in the stimulation of bile acid synthesis and fecal sterol excretion [8, 9]. We have also demonstrated that FCE reduces hepatic triglycerides in rats fed a high-cholesterol diet [9, 10]. Additionally, our previous study indicated that FCE ameliorates hepatic steatosis induced by the exposure to xenobiotics [9]. However, FCE may alter the metabolism of triglycerides, but the regulation mechanism is still unclear.

Foods containing soybean are commonly consumed in Asian countries, and eating such foods is associated with a lower risk of developing cardiovascular disease [11]. Many studies have demonstrated the beneficial effects of soy protein isolate (SPI), including a reduction in levels of blood cholesterol and triglycerides, particularly in hypercholesterolemic subjects, and a reduction in liver triglycerides and cholesterol in rats [12–16]. Ascencio et al. demonstrated that soy protein prevented the development of hepatic steatosis through the reduction in the hepatic gene expression of lipogenic enzymes in rats [12]. However, various mechanisms underlying the beneficial effects of SPI have been proposed [17]. 

In this study, we first compared the effects of FCE with those of SPI on the metabolism of triglycerides and cholesterol in rats in order to evaluate the practical benefits of FCE. We found that FCE and SPI had a similar effect on hepatic and serum cholesterol and hepatic triglycerides; however, FCE showed a much more powerful induction of fecal sterols excretion, suggesting that FCE improves lipid metabolism through a different mechanism from that of SPI. To compare the mechanism and function of this beneficial effect, we investigated the alteration of lipid profile in the liver of rats and then performed hepatic gene expression analysis. Finally, in the second experiment, we examined global gene expression in FCE in contrast with control diet by using microarray analysis. We found a novel effect of FCE in ameliorating triglyceride metabolism.

## 2. Materials and Methods

### 2.1. Chemicals

Kits for quantification of cholesterol (T-Chol), triglycerides (Triglyceride E-test), and phospholipids (Phospholipids C-Test) were purchased from Wako Chemical Co. Ltd. (Osaka, Japan). Serum adiponectin (mouse or rat adiponectin) enzyme-linked immunosorbent assay (ELISA) kit was obtained from Otsuka Pharmaceutical (Tokyo, Japan). Megaprime DNA labeling system was purchased from Amersham (Tokyo, Japan). High Capacity cDNA Reverse Transcription Kit and Power SYBR Master Mix were obtained from Applied Biosystems (Carlsbad, CA, USA). Agilent Low RNA Input Fluor Linear Amp Kit was purchased from Agilent (Santa Clara, CA, USA).

### 2.2. Materials

FCE was prepared as described previously [8, 9]. Briefly, freshwater clams (*Corbicula *spp.) were steamed until the shells opened, and the edible portion was removed. The edible portion was minced, extracted with boiling water, and then filtered through an 80-mesh filter. The filtrate was spray-dried for use as the FCE. The FCE yield from the raw material was approximately 1.5% (w/w). FCE contains not only water-soluble materials but also approximately 35% water-insoluble materials. The approximate composition of the FCE was 59.8 g protein, 11.6 g carbohydrate, 4.8 g moisture, 18.2 g crude fat, and 5.6 g ash per 100 g of powder [18]. The amino acid profile of FCE has been compared with that of casein [18], and we concluded that the profiles were closely similar.

### 2.3. Animals and Diets

Four-week-old male Wistar rats with a body weight of about 100 g were obtained from Japan SLC (Hamamatsu, Japan). The animals were maintained at 23°C with a 12 h light (0800 to 2000) and 12 h dark (2000 to 0800) cycle. To accustom the rats to the experimental conditions, we fed them a commercial stock diet (5L37; Japan SLC Inc.) for 4 days and then a basal diet containing 20% casein for 3 days before dividing them into groups of 6 animals each. The composition of the experimental diets is shown in Table 1. Previously, we investigated the effect of FCE on hypercholesterolemia in rats [8–10]. In this study, we attempted to evaluate the potential beneficial effect of FCE for preventing lifestyle-related diseases. In the first experiment, the animals were fed a basal diet (control group), a basal diet supplemented with 30 g FCE per 100 g food (FCE group), or a basal diet supplemented with 19.9 g SPI per 100 g food (SPI group) for 2 weeks. In the second experiment, the animals were fed a basal diet (control group) or a basal diet supplemented with 30 g FCE per 100 g food (FCE group) for 2 weeks. FCE or SPI was added to the basal diet at the expense of casein, sucrose, and **α**-cornstarch (Table 1). Total energy and protein amounts were equilibrated between groups. The same amounts of dietary proteins were introduced in the diet equally because the protein fraction is a major component of FCE and has hypocholesterolemic function [10]. During the study, the rats were maintained in individual stainless-steel cages and had free access to the experimental diets and water. Feces were collected over the final 3 days of the experimental period and used for determining fecal sterols. All the experiments were performed in accordance with an animal protocol approved by the Review Board for Animal Ethics at Oita University (permission number E048001) and Nagoya University (permission number 2008071601).

### 2.4. Biochemical Analyses

Blood samples were collected from the tail vein. About 2.5 g of liver tissue was homogenized, and the lipids were extracted using a chloroform/methanol mixture (2 : 1, v/v) as described by Folch et al. [19]. Serum and hepatic cholesterol and triglyceride, hepatic phospholipids, and serum adiponectin levels were determined using a commercial kit. Hepatic fatty acid content was analyzed as follows. Tricosanoic acid was added to the extracted lipids to be used as an internal standard. The mixture was dried under a flow of dry nitrogen gas at 37°C. Lipids were converted to methyl ester by the addition of 0.5 mL 5% hydrogen chloride-methanol solution and incubation at 75°C for 3 h. The samples were injected in a 25 m × 0.25-mm SGE-BPX70 capillary column (SGE, Victoria, Australia) and analyzed using gas chromatography/mass spectrometry (GCMD-QP5000; Shimadzu Co., Kyoto, Japan). Fecal sterols were extracted using the method of Delaney et al. [20]. Fecal neutral sterols were analyzed as trimethylsilyl ester by gas chromatography/mass spectrometry (GC 6890 equipped with 5973MSD and a 30 m × 0.25 mm HP-5 MS capillary column; Agilent) with 5*α*-cholestane as the internal standard. Fecal bile acids were determined enzymatically by using the method of Sheltawy and Losowsky [21], with lithocholic acid as the standard.

### 2.5. Total RNA Extraction and RNA Analysis

Total RNA from liver tissue was isolated according to the method described by Chomczynski and Sacchi [22], and 20 *μ*g total RNA was subjected to northern blot hybridization. The cDNA clones of rat apolipoprotein (*apo*) *A-I*, rat *CYP7A1*, rat ATP-binding cassette subfamily member 5 (*ABCG5*), rat fatty acid synthase (*FAS*), rat acetyl-coenzyme A carboxylase alpha (*ACACA*), rat fatty acid-binding protein 2 intestinal (*FABP2*), rat fatty acid-binding protein 5 epidermal (*FABP5*), rat sterol regulatory element-binding protein 1 (SREBP-1), rat stearoyl-coenzyme A desaturase 1 (*SCD1*), and mouse *apoE* were labeled with the Megaprime DNA labeling system and used for hybridization. Specific hybridization was quantified using an image analyzer (BAS 2000; Fuji Film, Tokyo, Japan). In our previous studies, *apoE* mRNA level was not affected by the diet [8–10]. Moreover, the *apoE* mRNA level was not affected by any treatment used in this study (data not shown); thus, we used it as a normalization standard. For real-time quantitative reverse transcription-polymerase chain reaction (qRT-PCR) assay, cDNA was prepared using a High Capacity cDNA Reverse Transcription Kit following the manufacturer's instructions. The forward and reverse primer sequences used in the qRT-PCR assay were as follows: rat long-chain fatty acid elongase family member 6 (*ELOVL6*) forward primer, 5′-ACCCGAACTAGGTGATACG-3′; rat *ELOVL6* reverse primer, 5′-CCCAGCTACCATGTCTTTG-3′; rat *apoE* forward primer, 5′-TTGGTCCCATTGCTGACAGG-3′; rat *apoE* reverse primer, 5′-GGTAATCCCAGAAGCGGTTC-3′. qRT-PCR amplifications were performed in an ABI StepOne (Applied Biosystems) in a reaction mixture of 20 *μ*L, which contained 8 *μ*L 80 × diluted cDNA, 10 *μ*L 2 × Power SYBR Master Mix, and 200 nM of each primer. The qRT-PCR melting curve data were collected to check for PCR specificity. Expression was calculated using the standard curve method. In addition, the expression of the chosen genes was normalized to that of *apoE* as an internal control.

### 2.6. Estimation of the Transcription Rate of the *CYP7A1* Gene

To estimate the transcription rate of the *CYP7A1* gene, we used the method described by Ripperger and Schibler [23]. We quantified *CYP7A1* pre-mRNA levels by real-time RT-PCR as described above. The primers were designed within intron 5 of the *CYP7A1* gene. The cDNA was PCR-amplified in ABI StepOne (Applied Biosystems). The primers used were as follows: rat pre-mRNA of *CYP7A1* forward, 5′-GAGTAGTATTTGGGAGGGATC-3′; rat pre-mRNA for *CYP7A1* reverse, 5′-TGAATGTGTGTTTGCTGAGGC-3′. The relative amount of *CYP7A1 *pre-mRNA was normalized with *apoE* mRNA as measured by qRT-PCR.

### 2.7. Microarray Analysis

The total RNA from liver tissue of the control group (*n* = 6) and FCE group (*n* = 6) was pooled separately in identical amounts for analysis. Next, 500 ng of total RNA was used for fluorescently labeled cRNA synthesis with Agilent Low RNA Input Fluor Linear Amp Kit, by following the manufacturer's protocol. For hybridization, 0.75 *μ*g Cy3- or Cy5-labeled cRNA from the control and FCE groups was combined and hybridized to an Agilent 22 K rat oligo microarray according to the manufacturer's protocol (Hokkaido System Science Co. Ltd., Sapporo, Japan). The oligonucleotide microarray slides were scanned using an Agilent microarray scanner, and the gene expression profiles were analyzed with Agilent microarray software. Locally weighted scatter plot smoothing normalization and ratio (FCE/control) calculation were conducted using the GeneSpring software (Agilent, Santa Clara, CA). Genes were considered upregulated if they had a ratio (FCE/control) of more than 1.46 and downregulated if they had a ratio of less than 0.68. Network analysis and biofunction analysis were performed using the Ingenuity Pathway Analysis (IPA) software (Ingenuity Systems, Redwood City, CA, USA) with differentially expressed gene expression values. Biofunctions were filtered according to *P* values of <.001. Functional analysis of all differentially expressed genes was performed using the database for annotation, visualization, and integrated discovery (DAVID) resource (http://david.abcc.ncifcrf.gov/). The functional annotation clusters are shown as KEGG pathways that had a *P* value of less than .05.

### 2.8. Statistical Analysis

The significance of differences among values was analyzed by the Student's *t*-test or one-way analysis of variance and then by Tukey's multiple-range test. The analyses were performed using SPSS version 10.0 (SPSS Japan, Tokyo, Japan). When the *P* value was less than .05, differences were considered significant. Values are expressed as means ± SEM.

## 3. Results

### 3.1. Body Weight Gain, Food Intake, Organ Weight, and Serum Parameters

Body weight was slightly decreased in the SPI group, but the food intake was not affected by the diets (Table 2). Liver weight was lower in the SPI group than in the control and FCE groups. Epididymal adipose tissue weight was not affected by the diets. The FCE and SPI groups showed significantly reduced serum cholesterol levels (Table 2). Serum triglycerides tended to be lower in the FCE group than in the control and SPI groups; however, the change was not significant (Table 2). SPI elevated the serum level of adiponectin, and FCE showed a tendency to increase this parameter, but this was not significant (Table 2). 

### 3.2. FCE Improved Cholesterol Metabolism Similar to SPI

**Figure 1:**
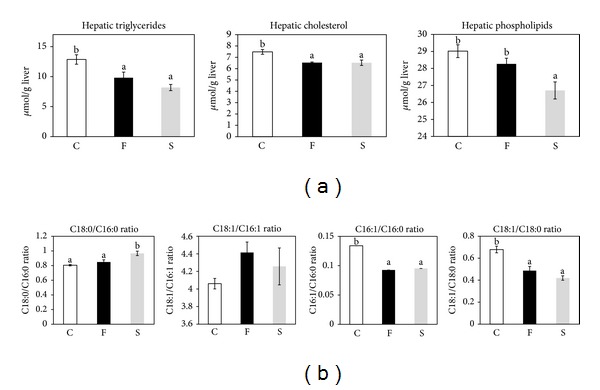
Hepatic lipids, index of hepatic elongation of palmitate and palmitoleate, and desaturation index of palmitate and stearate in rats fed FCE and SPI. In rats fed control diet, FCE, or SPI, hepatic lipids were extracted, and triglycerides, cholesterol, and phospholipids were quantified using commercial kits (a). Extracted hepatic lipids were converted to methyl ester, and the fatty acid profile was analyzed using gas chromatography/mass spectrometry. Then, fatty acid ratios were calculated to estimate the elongation of palmitate and palmitoleate (C18:0/C16:0, C18:1/C16:1) and desaturation of palmitate and stearate (C16:1/C16:0, C18:1/C18:0) (b). The statistical significance of differences among values was analyzed by ANOVA and then by Tukey's multiple-range test. Values with different letters indicate a statistically significant difference, *P* < .05. Each value is expressed as the mean ± SEM for 6 rats in each group (control (C), FCE (F), SPI (S)).

**Figure 2:**
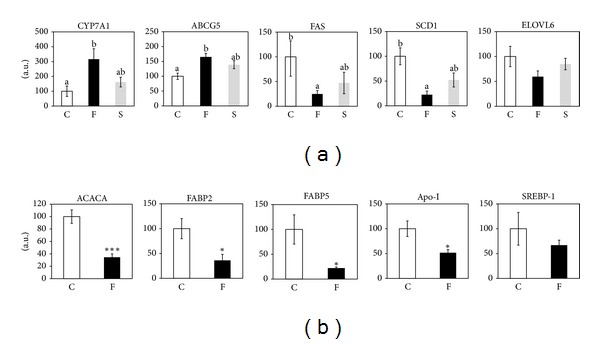
Hepatic levels of the genes involved in cholesterol metabolism, bile acid biosynthesis, and transport and biosynthesis of fatty acids in rats fed FCE and SPI. In the first experiment, the levels of mRNA for *CYP7A1*, ABCG5, FAS, SCD1, and ELOVL6 were measured by northern blotting. Each value is expressed as the mean ± SEM for 6 rats in each group (control (C), FCE (F), SPI (S)). The statistical significance of differences among values was analyzed by ANOVA and then by Tukey's multiple-range test. Values with different letters indicate a statistically significant difference, *P* < .05 (a). In the second experiment (control group (C) versus FCE group (F)), the levels of mRNA for ACACA, FABP2, FABP5, *apo A*-*I*, and SREBP-1 were measured by northern blotting. Each value is expressed as the mean ± SEM for 6 rats in each group. The statistical significance of differences among values was analyzed by Student's *t*-test. In each graph, * and *** indicate statistically significant difference at *P* < .05 and *P* < .001, respectively (b). *ApoE* was used as a normalization standard, as its levels were not significantly changed in our experiments. FCE, freshwater clam extract; SPI, soy protein extract; *CYP7A1*, cytochrome P-450 7A1; FAS, fatty acid synthase; ACACA, acetyl-coenzyme A carboxylase alpha; SCD1, stearoyl-coenzyme A desaturase 1; FABP2, fatty acid-binding protein 2 intestinal; FABP5, fatty acid-binding protein 5 epidermal; SREBP-1, sterol regulatory element-binding protein 1; ABCG5, ATP-binding cassette subfamily G, member 5; ELOVL6, elongation of very long-chain fatty acids protein 6; *Apo A*-*I*, apolipoprotein *A*-*1*.

The hepatic cholesterol-lowering effect of FCE was similar with that of SPI (Figure 1(a)). FCE induced fecal excretion of neutral and acidic steroids much more potently than SPI (Table 2). From these data, we inferred that FCE had a more powerful effect on cholesterol catabolism than SPI. We confirmed that FCE induced hepatic *CYP7A1* gene expression, the rate-limiting enzyme for bile acid biosynthesis (Figure 2(a)) [8]. Although SPI was reported to induce *CYP7A1* gene expression, the induction was smaller than that by FCE (Figure 2(a)). The transcription rate of the *CYP7A1* gene was evaluated by measuring the pre-mRNA level of the *CYP7A1* gene, and we found that FCE induced hepatic *CYP7A1* gene transcription more than SPI (Table 2). As we reported [10], FCE induced hepatic *ABCG5* gene expression, and SPI tended to increase this gene expression; however, the change was not significant (Figure 2(a)).

### 3.3. FCE May Ameliorate Triglyceride Metabolism through the Inhibition of Lipogenesis in the Liver

Hepatic triglycerides were lower in rats fed both FCE and SPI (Figure 1(a)). C16:0/C16:1 fatty acid elongation and monodesaturation of C18:0 and C16:0 were previously reported to be related to the regulation of fatty acid metabolism and lipogenesis in the liver [24]. Thus, we evaluated changes in the elongation and Δ9-desaturation of these fatty acids by measuring the fatty acid profile in rat liver. The C18:0/C16:0 ratio was higher in the SPI group than in the control and FCE groups, and the C18:1/C16:1 ratio was not affected in either the SPI or FCE group (Figure 1(b)). However, the 16 : 1/16 : 0 and 18 : 1/18 : 0 ratios were significantly lower in the FCE and SPI groups than in the control group (Figure 1(b)). To gain a deeper insight into the regulation of lipogenesis in the liver, we measured the mRNA levels of *SCD1* and *ELOVL6*, which are involved in the Δ9-desaturation of C16:0 and C18:0 and elongation of C16:0 and C16:1, respectively. Although *ELOVL6* mRNA level tended to be lower in the SPI and FCE groups, the differences were not significant (Figure 2(a)). *SCD1* mRNA level was significantly decreased by FCE, but not by SPI (Figure 2(a)). Downregulation of *SCD1* mRNA level was in accordance with the C18:1/C18:0 and C16:1/C16:0 ratios (Figures 1(b) and 2(a)). Moreover, gene expression of *FAS*, a lipogenic multienzyme, was significantly suppressed by FCE and tended to be reduced in SPI, although this was not significant (Figure 2(a)). Altogether, we conclude that both FCE and SPI reduced hepatic triglycerides through a similar mechanism, which involves the inhibition of the expression of lipogenic genes.

### 3.4. Analysis of Hepatic Transcriptome in Rats Fed FCE Revealed the Alteration of Gene Expression in Fatty Acid Synthesis and Transport

To gain an insight into the mechanism of the effect of FCE on lipid metabolism in the liver, we used oligonucleotide microarray to investigate gene expression in rats fed the basal and FCE diets in a second animal experiment. The microarray results showed that FCE downregulated the expression of 210 genes and upregulated the expression of 182 genes. Using IPA, analysis of biofunctions indicated significant changes in several functions, particularly lipid and drug metabolism (Table 3). Analysis of pathways by using DAVID indicated an induction of bile acid biosynthesis and an inhibition of polyunsaturated fatty acid and cholesterol biosynthesis (Table 3). Further analysis of subcategories of lipid metabolism by IPA indicated that fatty acid synthesis and transport and cholesterol and bile acid biosynthesis had *P* values less than .05 (see Table S1 in Supplementary Material available online at http://dx.doi.org/10.1155/2013/830684.) To validate the significance of the microarray results, we selected appropriate genes in subcategories in Supplementary Table S1 and measured liver gene expression (Figure 2(b)). The changes in gene expression observed in Figure 2(a) were also validated in this experiment (data not shown). We found that the mRNA level of *ACACA*, also involved in fatty acid biosynthesis, was notably reduced by FCE and that the expression of *FABP2* and *FAPB5* was reduced by FCE (Figure 2(b)). *Apo A-I* mRNA, which is involved in lipid transport, was also suppressed by FCE. These results obtained with northern blotting and real-time RT-PCR were in agreement with the microarray data (Supplementary Information, Table S1). Although *SREBP-1*, a key transcription factor involved in lipogenesis, tended to decrease in the FCE group, the difference was not significant in our experiment (Figure 2(b)).

## 4. Discussion

We previously demonstrated that an FCE diet improves blood cholesterol in rats with hypercholesterolemia [8, 9]. Our previous results suggest that this is due to an increase in the fecal excretion of bile acids and the cholesterol degradation to bile acid through enhancement of *CYP7A1* gene expression in the liver. Moreover, hepatic lipids were reduced by FCE in rats fed a high-cholesterol diet [9]. SPI affects serum triglycerides and cholesterol through several mechanisms, including a reduction in the insulin/glucagon ratio and a reduction in intestinal cholesterol absorption and bile acid uptake, conversion of cholesterol to bile acids [12, 25]. SPI also decreases hepatic triglyceride and cholesterol levels. As it was suggested that SPI has a similar effect to FCE on lipid balance, in the present study, we compared the ability of FCE to improve the metabolism of triglycerides and cholesterol in rat liver to that of SPI and investigated the mechanism of FCE effect.

FCE and SPI improved serum total cholesterol in a similar fashion, and FCE tended to reduce serum triglyceride level (Table 2). Since both diets improved liver cholesterol similarly, we compared the effect of FCE on cholesterol catabolism with that of SPI. We found that fecal excretion of both bile acids and neutral sterols and the hepatic expression of *CYP7A1* and *ABCG5* were importantly elevated in rats fed FCE only, as reported previously (Table 2 and Figure 2(a)) [10]. SPI has been reported to increase *CYP7A1* gene expression and fecal sterol [26], although in this study, this increase was not significant for SPI (Table 2 and Figure 2(a)). *CYP7A1* mRNA was also reflected at the transcription level by measuring the pre-mRNA level of this gene (Table 2). The main transcription factors, which were involved in the regulation of this gene, such as small heterodimer partner, liver X receptor, and farnesoid X receptor, were not altered by FCE (data not shown), as reported previously [9]. From the above results, we conclude that the increase in both *CYP7A1* and *ABCG5* mRNA levels can explain the increase in fecal excretion of acidic and neutral sterols in the FCE group. 

Serum triglycerides tended to be lower in FCE (Table 2), although the differences were not significant. In rats fed FCE and SPI, we also observed a significant decrease in hepatic triglycerides (Figure 1(a)). C16:0/C16:1 fatty acid elongation and monodesaturation of C18:0 and C16:0 were related to fatty acid metabolism regulation and lipogenesis in the liver [24]. SCD1 is an important enzyme that participates in Δ9-desaturation of palmitate and stearate [27]. ELOVL6 catalyzes the elongation of palmitate and palmitoleate and thus plays a central role in the *de novo* synthesis of long-chain saturated and monounsaturated fatty acids [28]. *ELOVL6* and *SCD1* genes are related to hepatosteatosis, obesity, and insulin resistance, all of which are associated with metabolic syndrome [24, 29]. Thus, we investigated the C16:0/C16:1 fatty acid elongation (regulated by ELOVL6) and monodesaturation of C18:0 and C16:0 (regulated by SCD1) by evaluating hepatic fatty acid composition in rats fed FCE or SPI. The results revealed that Δ9-desaturation but not elongation may be inhibited by FCE and SPI (Figure 1(b)), which correlated well with mRNA levels of *ELOVL6* and *SCD1* (Figure 2(a)). It has been reported that knockdown of SCD1 leads to the reduction of liver triglyceride, plasma triglycerides and VLDLs, and the increase insulin sensitivity [30, 31]. Downregulation of *SCD1* expression by both FCE and SPI would be responsible for the changes in fatty acid composition in the rat liver. Although previous studies indicated that feeding soy protein reduced SCD 1 gene expression and improved insulin sensitivity [32, 33], the effect of FCE on insulin sensitivity and diabetes has not been determined. Moreover, lipogenesis-related *FAS* gene expression was downregulated in FCE and tended to be reduced in SPI (Figure 2(a)). SPI can reduce the expression of several genes associated with lipogenesis, such as *SREBP-1*, *FAS*, and malic enzyme [12, 17]. Altogether, we conclude that FCE exerts an important effect on the metabolism of triglycerides in the liver in a similar manner to SPI, which involves inhibition of lipogenic gene expression and results in the reduction of hepatic triglycerides. FCE contains fats and proteins, and the FCE protein profile is similar to that of casein. Thus, we supposed that FCE would act through a different mechanism from that of SPI.

Moreover, transcriptome analysis of gene expression by IPA revealed that synthesis of fatty acids (*FAS*, *ACACA*, and *SCD1*) was downregulated in FCE (Table 3 and Supplementary Information, Table S1). We confirmed that the gene expression of *ACACA*, involved in the rate-limiting step of fatty acid synthesis, was significantly reduced in FCE and correlated with the *FAS* mRNA level (Figure 2(b)). This suggests that fatty acid synthesis-related gene expression is inhibited by FCE, which may result in decrease in triglyceride assembly.

FABPs have been linked to lipid-related diseases such as hyperlipidemia [34, 35]. Gene expression analysis with IPA revealed that transport of fatty acids (*FABP2*, *FABP5*) was downregulated in FCE (Table 3 and Supplementary Information, Table S1). Although little is known about the function of FABP2 in the liver, there is evidence that Ala54Thr polymorphism of *FABP2* gene has a role in insulin resistance and obesity [34]. In addition, *FABP2* gene expression was upregulated in steatohepatitic mice, and this elevation was correlated with an increase in long-chain fatty acid uptake [36]. *FABP5* knockout mice showed an improvement in plasma triglycerides and cholesterol, as well as insulin resistance [35]. From these results, we conclude that the hepatic triglyceride-lowering effect of FCE would be explained by changes in gene expression during fatty acid synthesis (*FAS*, *ACC*, and *SCD1*) and fatty acid signaling (*FABP2*, *FABP5*). Further investigations are needed to elucidate this possible mechanism.

Adiponectin is intimately involved in the improvement of insulin resistance and lipid accumulation [37]. As a consequence, serum adiponectin exerts a beneficial effect on metabolic syndrome [37]. Serum adiponectin was enhanced by SPI and tended to be elevated in FCE; however, this was not significant (Table 2). SPI and high-cholesterol diet supplemented with FCE have been demonstrated to increase serum adiponectin [10, 38]. 

IPA network analysis revealed a putative mechanism through which *SREBP-1* downregulation could affect the expression of multiple genes associated with the synthesis of bile acids, cholesterol, and fatty acids (Supplementary Information, Figure S1). The mRNA level of *SREBP-1* tended to decrease in FCE, although the difference was not significant (Figure 2(b)). SREBP is synthesized in its precursor form and inserted into the endoplasmic reticulum membrane. Then, it is activated by proteolysis in response to the sterol status [39]. A recent report showed that SREBP-1c processing is downregulated by insulin [40]. Thus, we believe that FCE may be involved in the regulation of SREBP-1 processing. We are now investigating the processing. We at present hypothesize that SREBP-1 would be associated with the regulation of lipid metabolism by FCE.

## 5. Conclusion

In this study, we attempted to characterize the effect of FCE on lipid metabolism, particularly on the metabolism of triglycerides in the rat liver, in contrast with the effect of SPI. In doing so, we found potential benefits of FCE for ameliorating metabolic syndrome owing to its ability to improve the levels of blood and hepatic cholesterol and hepatic triglycerides. FCE has a powerful effect on cholesterol catabolism, as evidenced by the induction in the expression of genes related to cholesterol catabolism, and excretion of fecal sterols. FCE displayed an effect in improving metabolism of triglycerides similar to SPI, through the suppression of gene expression related to lipogenesis. Our data brought evidence that fatty acid Δ9-desaturation may have an important role in ameliorating hepatic triglycerides by FCE. Global analysis of hepatic gene expression proposed that SREBP-1 would be a central regulator of the metabolism of triglycerides and cholesterol in rats fed FCE. We currently perform further experiments in which the effect of FCE on protein level and activity of SREBP is studied. Although our results support the conclusion that FCE would have potential benefits for preventing metabolic syndrome, further studies are needed to indicate direct evidence for metabolic syndrome, such as the effect on obesity, diabetes, and insulin resistance.

## Supplementary Material

Subcategories of biofunctions related to the metabolisms of fatty acid and cholesterol were affected by FCE (Table S1).
Schematic illustration of the hypothetical mechanism of FCE ameliorative action on cholesterol and fatty acid metabolism (Figure S1).

## Figures and Tables

**Table 1:** Composition of the experimental diets.

	Control group^a^	FCE group^a^	SPI group^a^
	(g/kg)	(g/kg)	(g/kg)
Casein^b^	200	—	—
FCE^c^	—	300	—
SPI^d^	—	—	199
A-cornstarch	468.7	402	469.3
Sucrose	234.3	201	234.7
Corn oil	50	50	50
Choline chloride	2	2	2
AIN-93G-MX mineral mixture	35	35	35
AIN-93-VX vitamin mixture	10	10	10

^a^Control group: the basal diet; FCE group: freshwater clam extract diet; SPI group: soy protein isolate diet.

^b^Protein content: 89.6%.

^c^Protein content: 59.8%.

^d^Protein content: 90.0%.

**Table 2:** Effect of freshwater clam extract and soy protein isolate on body weight gain, food intake, organ weight, serum parameters, fecal steroids, and hepatic CYP7A1 transcription rate in rats^a^.

	Control group^b^	FCE group^b^	SPI group^b^
	Mean ± SEM	Mean ± SEM	Mean ± SEM
Body weight gain (g)	71.3 ± 3.4^ab^	77.3 ± 2.9^b^	64.8 ± 2.2^a^
Food intake (g/d)^c^	14.7 ± 0.6	13.3 ± 0.5	14.1 ± 0.5
Relative organ weight (g/100 g of body weight)			
Liver	3.40 ± 0.08^b^	3.44 ± 0.06^b^	2.66 ± 0.04^a^
Epididymal adipose tissue	1.52 ± 0.05	1.37 ± 0.05	1.10 ± 0.24
Serum lipids (mmol/L)			
Total cholesterol	2.28 ± 0.10^b^	1.56 ± 0.06^a^	1.56 ± 0.04^a^
Triglyceride	0.65 ± 0.04	0.57 ± 0.03	0.65 ± 0.02
Serum adiponectin (ng/mL)	4426 ± 293^a^	5562 ± 476^a^	7212 ± 310^b^
Dry fecal weight (g for 3d)^d^	1.2 ± 0.1^a^	3.2 ± 0.3^c^	1.7 ± 0.0^b^
Fecal steroids (*μ*mol/3 days)			
Total neutral sterol	29.5 ± 3.6^a^	119 ± 10^b^	42.5 ± 2.8^a^
Total bile acids	36.3 ± 4.4^a^	247 ± 18^b^	51.7 ± 3.2^a^
Hepatic transcription rate (arbitrary units)^e^			
CYP7A1	100 ± 19^a^	303 ± 47^b^	152 ± 26^a^

^a^Each value is the mean with its standard error for 6 rats in each dietary group. The statistical significance of differences among values was analyzed by ANOVA and then by Tukey's multiple-range test. Values in a row with different letters indicate a statistically significant difference, *P* < .05.

^b^Control group: basal diet; FCE group: freshwater clam extract-supplemented diet; SPI group: soy protein isolate-supplemented diet.

^c^Food intake was measured from day 2 to day 3.

^d^Feces were collected over the final 3 days of the experimental period.

^e^Transcription rate was estimated by the pre-mRNA level.

**Table 3:** Various biofunctions and pathways were altered in the rat liver by the FCE diet compared with control.

Category	*P* value	Gene	Upregulated genes	Downregulated genes
Biofunctions^a^				
Lipid metabolism	1.55*E* − 13	83	42	41
Small molecule biochemistry	1.55*E* − 13	103	55	48
Vitamin and mineral metabolism	9.80*E* − 13	34	18	16
Drug metabolism	2.88*E* − 10	26	21	5
Molecular transport	5.96*E* − 07	64	34	30
Nucleic acid metabolism	1.29*E* − 06	16	6	10
Carbohydrate metabolism	1.55*E* − 04	39	17	22
Cell death	1.57*E* − 04	21	19	2
Amino acid metabolism	7.70*E* − 04	17	15	2
Cell cycle	1.21*E* − 03	10	7	3
Cellular assembly and organization	1.21*E* − 03	9	2	7
Gene expression	1.21*E* − 03	15	11	4
Cellular function and maintenance	1.31*E* − 03	7	6	1
Cellular compromise	3.54*E* − 03	9	6	3
Cellular growth and proliferation	4.16*E* − 03	4	3	1
Cell-to-cell signaling and interaction	6.91*E* − 03	7	4	3
KEGG pathways^b^				
Polyunsaturated fatty acid biosynthesis	5.95*E* − 06	7	0	7
Metabolism of xenobiotics by cytochrome P450	7.81*E* − 06	12	12	0
Biosynthesis of steroids	8.09*E* − 05	7	0	7
C21-steroid hormone metabolism	2.79*E* − 04	5	3	2
Androgen and estrogen metabolism	3.08*E* − 04	9	6	3
PPAR signaling pathway	1.92*E* − 03	9	1	8
Linoleic acid metabolism	3.14*E* − 03	6	6	0
Alanine and aspartate metabolism	3.61*E* − 03	6	3	3
Maturity onset diabetes of the young	7.55*E* − 03	5	0	5
Pyruvate metabolism	1.03*E* − 02	6	1	4
Bile acid biosynthesis	2.68*E* − 02	5	4	1
Glutathione metabolism	3.48*E* − 02	5	4	1
Carbon fixation	3.56*E* − 02	4	1	3
Nitrogen metabolism	3.56*E* − 02	4	2	2

We compared the effect of the FCE diet on hepatic gene expression with that of control by using oligonucleotide microarray.

^a^Differentially expressed gens list from microarray experiment was imported in Ingenuity pathway analysis, filtered by selecting only genes expressed in rat liver. Significance values expressed as *P* values were calculated using a right-tailed Fisher's exact test. Biofunctions that were significantly changed were determined.

^b^We conducted KEGG pathway analysis by importing a list of differentially expressed genes in DAVID (database for annotation, visualization, and integrated discovery). Significance values expressed as *P* values were calculated using a modified Fisher exact probability test (EASE score).
