# Supplementary material for: Freshwater Clam Extract Ameliorates Triglyceride and Cholesterol Metabolism through the Expression of Genes Involved in Hepatic Lipogenesis and Cholesterol Degradation in Rats
Source: Evid Based Complement Alternat Med. 2013 Feb 12;2013:830684. doi: 10.1155/2013/830684 (PMC3583048; doi:10.1155/2013/830684)
Supplement: Supplementary file 1 — Subcategories of biofunctions related to the metabolisms of fatty acid and cholesterol were affected by FCE (Table S1). Schematic illustration of the hypothetical mechanism of FCE ameliorative action on cholesterol and fatty acid metabolism (Figure S1). [file 830684.f1.pdf]

Table S1. Subcategories of biofunctions related to the metabolisms of fatty acid and cholesterol were affected by FCE

Gene description	Fold change[b]	P value
**Genes related to synthesis of fatty acids[a]**		**5.77E-05**
Fatty acid synthase	-11.2	
Fatty acid desaturase 2	-3.79	
Stearoyl-Coenzyme A desaturase 1	-3.61	
Fatty acid desaturase 1	-3.24	
ATP citrate lyase	-2.75	
Acetyl-coenzyme A carboxylase alpha	-2.62	
Stearoyl-Coenzyme A desaturase 2	-2.21	
Sterol regulatory element binding factor 1	-2.03	
Dihydrolipoamide S-acetyltransferase (E2 component of pyruvate dehydrogenase complex)	-1.71	
Lipase, hepatic	-1.60	
CD14 antigen	2.01	
Acyl-CoA synthetase medium-chain family member 3	2.39	
**Genes related to transport of fatty acids**		**7.25E-03**
Fatty acid binding protein 5, epidermal	-4.38	
Fatty acid binding protein 2, intestinal	-3.23	
Carnitine O-octanoyltransferase	1.58	
**Genes related to biosynthesis of cholesterol**		**2.37E-06**
Glucose-6-phosphate dehydrogenase X-linked	-3.64	
Sterol regulatory element binding factor 1	-2.03	
Cytochrome P450, subfamily 51	-1.94	
Phenylalkylamine Ca2+ antagonist (emopamil) binding protein	-1.72	
7-dehydrocholesterol reductase	-1.70	

Farnesyl diphosphate synthase	-1.64	
Insulin induced gene 1	-1.60	
Apolipoprotein A-I	-1.51	
**Genes related to transport of cholesterol**		**1.17E-02**
Lipase, hepatic	-1.60	
Apolipoprotein A-I	-1.51	
ATP-binding cassette, sub-family G (WHITE), member 5	3.39	
Cytochrome P450, family 7, subfamily a, polypeptide 1	4.16	
**Genes related to biosynthesis of bile acids**		**2.99E-03**
Hydroxy-delta-5-steroid dehydrogenase, 3 beta- and steroid delta-isomerase 7	1.71	
Aldo-keto reductase family 1, member D1	1.95	
Cytochrome P450, family 7, subfamily a, polypeptide 1	4.16	

We compared FCE diet effect on hepatic gene expression with control using oligonucleotide microarray.

[a]Differentially expressed gene list from microarray experiment were imported in Ingenuity pathway analysis®, filtered by selecting only genes expressed in rat liver. Significance expressed as *P*-values were calculated using the right-tailed Fisher's exact test. Subcategories of biofunctions significantly changed were obtained.

[b]Fold changes in gene expression between FCE group and control group in microarray experiment are shown.

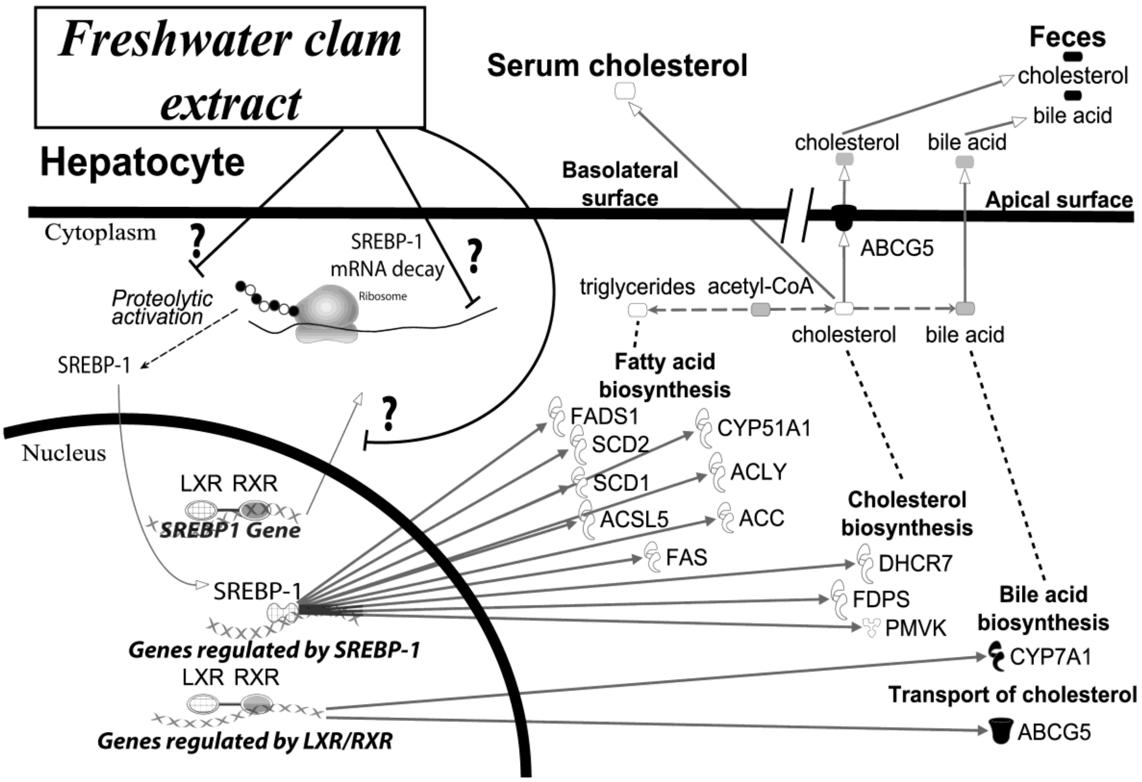

Figure S1. Schematic illustration of the hypothetical mechanism of FCE ameliorative action on cholesterol and fatty acid metabolism

IPA (Ingenuity Systems, www.ingenuity.com) was used to identify the SREBP-1 signaling network in rats fed FCE. This network is centered on the signaling protein SREBP-1. The downregulation of SREBP-1 may cause the downregulation of gene expression related to fatty acid biosynthesis and cholesterol biosynthesis. However, the expression of genes involved in bile acid biosynthesis and the transport of cholesterol were upregulated by FCE, although network analysis did not uncover a clear mechanism for this regulation. SREBP-1 and other unknown signaling proteins may be involved in FCE action in improving serum and hepatic cholesterol levels. White, black, and grey icons indicate downregulated, upregulated, and unknown amounts of lipids and gene expression in the FCE group, respectively. Icons filled

with crossed lines show that gene expression was not changed. Filled and open arrowheads describe the regulation of gene expression and translocation, respectively. Broken grey lines depict enzymatic reactions, and T bars describe the inhibition of cellular functions. Broken black lines indicate the relationships between lipid metabolism-related gene expression and lipid levels in the liver. FCE, freshwater clam extract; IPA, Ingenuity Pathways Analysis; SREBP-1, sterol regulatory element-binding protein 1; LXR, liver X receptor; RXR, retinoid X receptor; FADS1, fatty acid desaturase 1; SCD1/2, stearoyl-coA desaturase 1/2; ACSL5, acyl-CoA synthetase long-chain family member 5; ACC, acetyl-coA carboxylase; ACLY, ATP citrate lyase; PMVK, phosphomevalonate kinase; FDPS, farnesyl diphosphate synthase; DHCR7, 7-dehydrocholesterol reductase; ABCG5, ATP-binding cassette, sub-family G, member 5; CYP51A1, cytochrome P450, family 51, subfamily A, polypeptide 1; CYP7A1, cytochrome P450, family 7, subfamily A, polypeptide 1.
